# The Interstitial Lymphatic Peritoneal Mesothelium Axis in Portal Hypertensive Ascites: When in Danger, Go Back to the Sea

**DOI:** 10.4061/2010/148689

**Published:** 2010-10-05

**Authors:** M. A. Aller, I. Prieto, S. Argudo, F. de Vicente, L. Santamaría, M. P. de Miguel, J. L. Arias, J. Arias

**Affiliations:** ^1^Surgery I Department, School of Medicine, Complutense University of Madrid, 28040 Madrid, Spain; ^2^Surgery Department, School of Medicine, Autonoma University of Madrid, 28046 Madrid, Spain; ^3^General Surgery Unit, Sudeste Hospital, Arganda del Rey, 28500 Madrid, Spain; ^4^Cellular Biology and Morphological Sciences Department, School of Medicine, Autonoma University of Madrid, 28046 Madrid, Spain; ^5^Cell Engineering Laboratory, La Paz Hospital, Autonoma University of Madrid, 28046 Madrid, Spain; ^6^Neurosciences Unit, Psychobiology Department, School of Psychology, University of Oviedo, 33003 Oviedo, Asturias, Spain

## Abstract

Portal hypertension induces a splanchnic and systemic low-grade inflammatory response that could induce the expression of three phenotypes, named ischemia-reperfusion, leukocytic, and angiogenic phenotypes.During the splanchnic expression of these phenotypes, interstitial edema, increased lymph flow, and lymphangiogenesis are produced in the gastrointestinal tract. Associated liver disease increases intestinal bacterial translocation, splanchnic lymph flow, and induces ascites and hepatorenal syndrome. Extrahepatic cholestasis in the rat allows to study the worsening of the portal hypertensive syndrome when associated with chronic liver disease. The splanchnic interstitium, the mesenteric lymphatics, and the peritoneal mesothelium seem to create an inflammatory pathway that could have a key pathophysiological relevance in the production of the portal hypertension syndrome complications. The hypothetical comparison between the ascitic and the amniotic fluids allows for translational investigation. From a phylogenetic point of view, the ancestral mechanisms for amniotic fluid production were essential for animal survival out of the aquatic environment. However, their hypothetical appearance in the cirrhotic patient is considered pathological since ultimately they lead to ascites development. But, the adult human being would take advantage of the potential beneficial effects of this “amniotic-like fluid” to manage the interstitial fluids without adverse effects when chronic liver disease aggravates.

## 1. Introduction

It has been proposed that low-grade inflammation related to portal hypertension (PH) switches to high-grade inflammation with the development of severe and life-threatening complications when associated with chronic liver disease [1].

 It is accepted that the underlying central theme in low-grade portal hypertensive inflammation is the disturbance in splanchnic and systemic hemodynamics [1, 2]. This splanchnic and systemic hemodynamic response would be aggravated during the progression of the chronic liver disease [1, 2]. Thus, a critical state is produced in which the appearance of noxious factors during the progressive evolution of chronic liver disease would favor the development of a high-grade splanchnic and systemic inflammatory response [1, 3, 4]. 

In the current paper, we have considered that portal hypertensive syndrome evolves in three stages of increasing severity during which a body hydrosaline decompensation of splanchnic origin is developed. This loss of hydrosaline body homeostasis is fundamentally produced by PH, although it is aggravated if liver disease is associated. From a histological and anatomical point of view, we have hypothesized that, firstly, the splanchnic interstitial space would be impaired, after that the mesenteric lymphatic system would be disturbed, and finally, the mesothelial peritoneal cavity would be involved. In the following first section of the paper, we describe the evolution of PH when there are not complications, particularly without associated liver disease. In this case, the portal hypertensive syndrome induces hyperdynamic splanchnic and systemic circulation, mesenteric venous vasculopathy, bacterial translocation to the mesenteric lymph nodes, and liver steatosis with metabolic syndrome. In the second and third sections, we explain how the evolution of PH is when mild or moderate liver insufficiency is associated.

## 2. The Role of Mast Cells in the Pathophysiology of the Portal Hypertensive Syndrome

PH induces a splanchnic and systemic low-degree inflammatory response that could be developed through the expression of three successive and overlapping phenotypes: ischemia-reperfusion phenotype, leukocytic phenotype, and angiogenic phenotype (Table 1). In turn, it has been already proposed that these phenotypes could represent the expression of trophic functional systems with increasing metabolic complexity [1, 5]. This hypothetical approach to the mechanisms that govern the systemic inflammatory response could be based on the increasing metabolic ability of the body over the successive phases of its evolution towards a splanchnic and systemic remodeling. Therefore, in the portal hypertensive patient, it could be considered that the body adapts the support (the trophic system) to the metabolic needs characteristic of each inflammatory phenotype. In turn, the metabolic ability of each inflammatory phenotype would be determined by the mechanism used for cellular energy production [1, 2, 5]. Mast cells strategically located close to blood vessels could be among the first to respond to the mechanical stimuli that initiate splanchnic inflammation in PH [2]. If so, the early hemodynamic alterations would favor an abnormal movement of fluids into the interstitial space which would subsequently induce the development of a splanchnic lymphatic hyperdynamic circulation. Moreover, mediators released by mast cell could participate in this lymphatic hyperdynamic circulation. When appropriately activated mast cells have the ability to produce vasoactive amines, enzymes, that is, proteases, cytokines, chemokines, and growth factors through degranulation [1, 2]. This plasticity of the mast cells can also show diverse responsiveness during the splanchnic inflammatory response evolution, and genetic and environmental factors can position them within a broad spectrum of functional responsiveness. If so, mast cells could successively participate in the expression of the three trophic functional systems which have been previously proposed as components of the inflammatory response [2]. 

Hyperdynamic circulation stands out among the splanchnic and systemic alterations related to PH [6, 7]. It has been suggested that the splanchnic and systemic vasodilatation is the initial step leading to the hyperdynamic syndrome or progressive vasodilatory syndrome [6, 8]. Multiple organ failure in portal hypertensive chronic liver disease is in large part attributable to this syndrome [7, 8]. Furthermore, hyperdynamic circulation could favor the initiation and maintenance of an inflammatory response. First, the pathological increase of the portal pressure that occurs in the hyperdynamic splanchnic circulation could favor a disturbed splanchnic venous flow with shear stress mediated by nonlaminar flow [6]. The disturbed or nonlaminar flow produced by low shear stress has profound effects on the biology of the vascular wall, particularly the vascular endothelium, and could stimulate inflammation [9]. Second, both the increase in blood flow speed and the opening of arteriovenous shunts that induce the splanchnic hyperdynamic circulation would reduce the oxygen tissue availability. This fact would produce tissue hypoxia and therefore the chronicity of the inflammatory response [2]. 

The ischemia-reperfusion phenotype secondary to the hyperdynamic syndrome could present oxidative and nitrosative stress with edema, which favors nutrition by diffusion through the inflamed tissues and organs. This hypothetical trophic mechanism has a low-energy requirement that does not require oxygen (ischemia-hypoxia), and to a certain degree oxygen cannot be adequately processed resulting in the production of reactive oxygen and nitrogen species (ROS/RNS) (reperfusion-reoxygenation). It is likely that the magnitude of tissues's and organ hypoxia is not uniformly distributed, which, in turn, would determine the heterogeneity of the inflammatory response. The venous hyperpressure associated with hypoxia could be an important trigger of the splanchnic mast cell activation. Degranulation of mast cells could result in the release of potent vasodilator and exudative mediators such as histamine, leukotrienes, cytokines, chemokines, and proteases [2]. During the expression of this phenotype, while the progression of the interstitial edema increases in the space between the epithelial cells and the capillaries, the lymphatic circulation is simultaneously activated (circulatory switch). Thus, the injured tissue would adopt an ischemic phenotype (hypoxia) [1, 2].

The expression of the leukocytic phenotype by the tissues and organs which have suffered ischemia-reperfusion is coupled with interstitial infiltration by inflammatory cells, including mast cells, and sometimes by bacteria. Symbiosis of the inflammatory cells and bacteria for extracellular digestion by enzyme release (fermentation) and by intracellular digestion (phagocytosis) could result in a hypothetical trophic capacity. Improper use of oxygen persists in this immune phase together with the presence of additional enzymatic stress. Compensation of the acute-phase response includes the production of positive acute-phase proteins that bind proteolytic enzymes and inhibitors of leukocyte and lysosomal proteolytic enzymes. Likewise, the natural inhibitors of matrix metalloproteinases (TIMPs) could promote antienzymatic stress [1, 2]. 

PH induces bacterial translocation which is defined as the migration of viable microorganisms from the intestinal lumen to the mesenteric lymph nodes and other extraintestinal organs and sites [10]. Bacterial overgrowth caused by delayed intestinal transit and mucosal hypoperfusion and oxidative damage, which increases intestinal permeability and induces the transmural passage of bacteria, has been considered among the crucial factors involved in bacterial translocation that exists in PH [11, 12]. 

**Figure 1:**
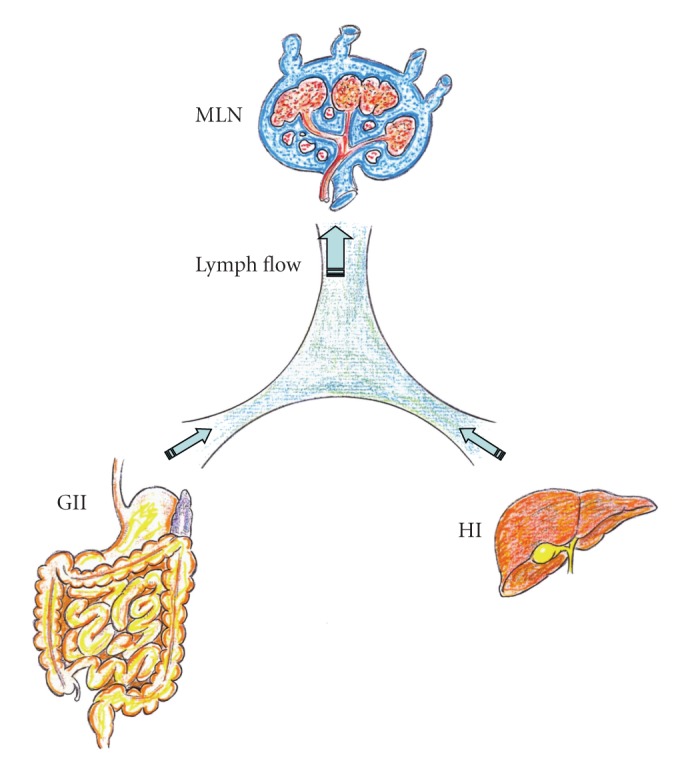
Splanchnic lymphatic flow resulting from the gastrointestinal interstitium (GII) and the hepatic interstitium (HI) is drained through the mesenteric lymph node (MLN) in physiological situations.

Furthermore, once activated by proinflammatory mediators, mast cells migrate to mesenteric lymph nodes through mesenteric lymphatic vessels and thus induce lymph node hypertrophy [13]. The mesenteric lymph nodes are key structures involved in the gut-associated lymphoid tissue (GALT) [14]. GALT has an important function in the maintenance of the intestinal mucosal integrity as well as in the control of mucosal inflammation. In particular, increased infiltration by mast cells in the small bowel and mesenteric lymph nodes in experimental PH suggests their involvement in the development of portal hypertensive enteropathy and therefore in bacterial translocation, through the release of their multiple inflammatory mediators [10, 13] (Figure 1).

The activation of the mast cells in the mesenteric lymph nodes in experimental PH would not only collaborate in the production of mesenteric adenitis, but also would constitute a source of mediators for the inflammatory response between the gastrointestinal tract and systemic blood circulation [15]. GALT constitutes the largest lymphoid organ of the body, and its activation in portal hypertensive enteropathy results in the release of several inflammatory mediators. These mediators would be transported by the intestinal lymph vessels to the pulmonary circulation—inducing an inflammatory phenotype—and later to the systemic circulation. The mesenteric lymph node circulation predominates, with respect to portal circulation, for transporting inflammatory mediators released in the intestinal wall in different conditions related to intestinal ischemia such as hemorrhagic shock or severe burns [16]. In this way, in other conditions that also produce intestinal ischemia, like PH, the mesenteric lymph represents a splanchnic vehicle for proinflammatory cytokines causing systemic effects as well [5]. 

It could be considered that remodeling by angiogenesis characterizes the third phenotype of the portal hypertensive inflammatory response (Table 1). Angiogenesis is defined as the growth of new vessels from preexisting ones [17]. Although the final objective of endothelial growth is to form new vessels for oxygen, substrates, and blood cells, other functions could also be carried out before the new vessels are formed. Particularly, it has been suggested that in the early phases of the inflammatory response, the new endothelial cells formed could have antioxidative, as well as antienzymatic stress properties. In case of ongoing oxidative and enzymatic stress, angiogenesis also plays a role in the tissue and organ remodeling [1, 18]. Since in PH the basic structural alteration found in the gastrointestinal tract is vascular and consists of increased size and number of the vessels, the very appropriate name of “hypertensive portal intestinal vasculopathy” has been proposed [19]. However, in addition to vascular alterations, histological evidence of nonspecific inflammation has been described in the gastroenteropathy associated with PH [19]. Therefore, angiogenesis plays a key role in development of portal hypertension (PHT) and represents a potential therapeutic target [20]. 

Chronic inflammatory infiltration found in the small bowel predominantly consists of mononuclear cells, and it is accompanied by atrophy, a decreased villous/crypt ratio, edema of the lamina propria, fibromuscular proliferation, and thickened muscularis mucosa [21, 22]. Since most of the aforementioned characteristics can be explained on the basis of increased levels of mast cell mediators [23], these cells could be involved in the pathogenesis of portal hypertensive chronic enteropathy [15]. Furthermore, in experimental portal hypertensive enteropathy, the increased degree of infiltration coexists with a higher vessel number in these intestinal layers. Indeed, cell number shows a positive and statistically significant correlation with the vascular diameter and total microvascular surface [24]. Splanchnic hyperemia, increased splanchnic vascularization, and the development of portal-systemic collateral circulation in experimental PH, all are partly vascular endothelial growth factor (VEGF-) dependent angiogenic processes [25, 26].

Long-term experimental PH shows persistent splanchnic alterations related to portal hyperpressure with the changes in the metabolism of lipids and carbohydrates that could be involved in the development of liver steatosis as well as in some of the manifestations described in the clinical metabolic syndrome [27–29]. Thus, in experimental chronic prehepatic PH, we have demonstrated the progressive fatty, triglyceride, and cholesterol infiltration of the liver combined with megamitochondria formation [28–30]. The mechanisms by which PH could induce liver steatosis are not fully understood. Nevertheless, the mechanisms that have been proposed in order to explain the pathophysiology also correspond with those expressed as result of a low-grade chronic inflammatory state [2, 31]. If so, mast cells would also be involved in the production of hypercholesterolaemia and liver abnormalities. Particularly, the mast cell activation syndrome should be considered a possible cause of hypercholesterolaemia and of hepatic abnormalities of unknown reason [32].

There is an intimate relationship between the immune and metabolic response systems that have many evolutionary underpinnings. Therefore, it is possible to imagine a situation in which common or overlapping pathways regulate both metabolic and immune functions through common key regulatory molecules and signaling systems [33]. Moreover, it is interesting to note that both adipose tissue and the liver have an architectural organization in which metabolic cells (adipocytes or hepatocytes) are in close proximity to immune cells (Kupffer cells or macrophages), and both have immediate access to a vast network of blood vessels [33]. Moreover, an important role of adaptive immunity has been demonstrated in NASH, a condition found in the majority of the patients who suffer from metabolic syndrome. In these patients, adipose tissue could activate T cells which in turn promote the recruitment and activation of macrophages in this tissue [34]. Since obesity fat-laden hepatocytes and adipocytes share common features, it is also possible that they may also share this immunologic T cell-mediated pathogenic mechanism. That is why it has been suggested that “obesity” neoantigens are involved in the production of a chronic low-grade inflammation of the adipose tissue and NASH in the development of metabolic syndrome [35]. 

Improper intestinal lymphatic function may also be present and be propagated by the chronic low-grade intestinal inflammation that PH presents. Intestinal lymphatic transport failure likely results in lymphatic immune dysfunction, leading to increased inflammation and increased release of inflammatory mediators, which could further impede the ability of lymphatics to function [36]. Lymphatic circulation, both intestinal and extraintestinal, is an important transporter of lipids and plays a role in metabolic function. Dysfunctional lymphatics result in lymph stasis and leakage which stimulates adipogenesis. Furthermore, failures in lymphatic transport can result in marked lipid accumulation throughout the body thus contributing to adipose tissue hyperplasia [36, 37]. The expansion of the perilymphatic adipose deposits also appears to occur with localized chronic inflammation and is considered metabolically essential for proper immune response and as a source of energy for immune activation and proliferation [38, 39].

## 3. The Role of Disturbed Splanchnic Lymph Drainage in the Portal Hypertensive Syndrome

Liver disease could be the most frequent factor for worsening portal hypertensive syndrome and therefore the splanchnic lymphatic function. Particularly, chronic liver disease and cirrhosis aggravate this syndrome exceedingly and favor lymphatic dysfunction [40] (Table 2). Therefore, it can be hypothesized that when PH is associated with mild or moderate liver disease, it would aggravate the hyperdynamic lymphatic splanchnic circulation, thus promoting the dilation of the lymphatic vascular system and probably a pathological lymphangiogenesis. Also, in this lymphatic condition, the mediators released by the splanchnic mast cells could participate.

Hepatic dysfunction related to fibrosis or cirrhosis would aggravate the grade of systemic inflammation characteristic of the chronic portal hypertensive syndrome. Hepatic dysfunction produces splanchnic and systemic hyperdynamic circulation which in turn favors lymphatic hyperdynamic circulation. Increased lymph flow is known to occur in diffuse abnormalities of liver architecture such as fibrosis and cirrhosis [41, 42]. The size and number of lymphatics are increased due to the excessive hepatic lymph production which is caused by disturbance of the microcirculation typical of PH [42]. Expansion of lymphatics is also a prominent feature of gastrointestinal inflammation. The dysregulation of lymphatics in turn, exacerbates, gastrointestinal disease [43]. During cirrhotic PH, dilation of esophageal and gastric lymph vessels may be related to the absorption of excess interstitial fluid [44]. However, the main features of liver decompensation in cirrhosis are ascites [45] hepatorenal syndrome [40, 46], and hepatic encephalopathy [40]. 

**Figure 2:**
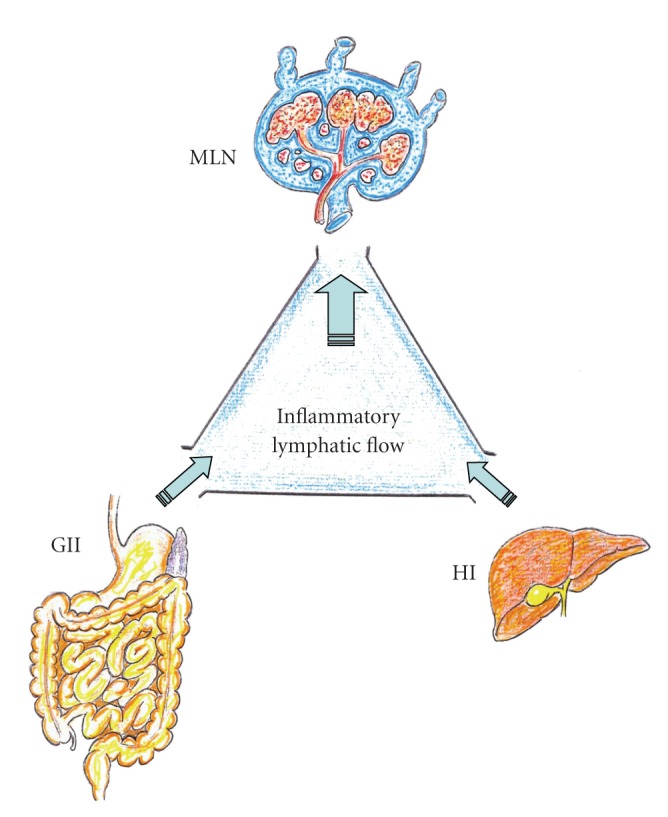
Chronic low-grade splanchnic inflammation in the portal hypertensive syndrome increases mesenteric lymphatic flow. GII: Gastrointestinal interstitium; HI: Hepatic interstitium; MLN: Mesenteric lymph nodes.

Ascites and hepatorenal syndrome are the major challenging complications of cirrhosis and PH that significantly affect the course of the disease [47]. Hepatorenal syndrome is a serious complication of end-stage disease, occurring mainly in patients with advanced cirrhosis and ascites who have marked circulatory dysfunction [46]. Although ascites can be observed in multiple diseases [48], it is most frequent due to cirrhosis with PH. The three major factors involved in the pathogenesis of ascites are PH, arterial vasodilation, and neurohormonal activation, all of them leading to sodium and water retention [47]. Arteriolar vasodilation causes underfilling of systemic arterial vascular space, with a decrease in the effective arterial blood volume. Consequently, baroreceptor-mediated activation of renin-angiotensin-aldosterone system (RAAS), sympathetic and parasympathetic nervous systems, and the release of antidiuretic hormone all aim at restoring normal circulatory function [47, 49–51]. On the other hand, splanchnic vasodilation increases splanchnic lymph production exceeding the lymph transportation system capacity and leads to lymph leakage into the peritoneal cavity [52]. Persistent renal sodium and water retention, alongside increased splanchnic vascular permeability in addition to lymph leakage into peritoneal cavity, plays the major role in a sustained ascites formation [50, 52]. Over time, the stasis of lymph flow in lymphatic channels of the intestine could lead to lymphangiectasia with an accompanying loss of proteins and lymphocytes [53]. In the past, it was considered that its treatment depended on a reduction of lymph formation indirectly, that is, dietary restriction of salt and water and diuretic drugs, or directly, that is, portosystemic shunt, portal decompression, or alternatively, an acceleration of an already rapid lymph return (peritoneovenous shunt) to match the high rate of lymph production. Conversely, factors that favor lymph formation (i.e., mineralocorticoids, exogenous salt) or inhibit lymph return (i.e., impaired lymphatic contractility) or central nervous hypertension, intensify lymph imbalance and worsen ascites [54] (Figure 2).

Other mechanisms proposed to be involved in ascites formation are based on hepatorenal reflex, secondary to a rapid increase in sinusoidal hepatic pressure [52, 55] and the splenorenal reflex-mediated reduction in renal vascular conductance, which exacerbates sodium and water retention in the kidneys. This may eventually contribute to renal dysfunction, and consequently ascites formation [56]. The development of ascites is a major complication of cirrhosis that induces an impaired quality of life and decreased survival. The most difficult patients to treat are those with refractory ascites, which are characterized by a lack of response to diuretic treatment [57]. 

Spontaneous bacterial peritonitis (SBP) is a frequent and severe complication of decompensated cirrhosis [58]. Bacterial translocation is the key mechanism in its pathogenesis, and the common causative microorganisms are gram-negative bacteria such as Escherichia coli and Klebsiella pneumoniae. Other less frequent causative microorganisms include pneumococci and streptococci [59]. SBP is often clinically unapparent and requires a high index of suspicion and almost always occurs in large-volume ascites in patients with liver cirrhosis. Abdominal pain can be continuous and is different from tense ascites. Ascitic polymorphonuclear leukocyte cell count is essential for diagnosis and management [58, 59]. However, the presence of fragments of bacterial DNA can be identified by a polymerase chain reaction (PCR-) based method and automated nucleotide sequencing in patients with culture-negative, nonneutrocytic ascites, indicating the existence of bacterial DNA (bactDNA) translocation [60]. The presence of bacterial DNA in blood and ascitic fluid in a significant number of patients with decompensated cirrhosis without bacterial infections may have an important role not only as a marker of bacterial translocation, but also as a short-term prognostic factor [60]. Paralytic ileus, hypotension, and hypothermia are seen in advanced illness, possibly resulting in renal failure (about one third of patients) or in death [50, 59]. Secondary bacterial peritonitis is an infrequent complication in cirrhotic patients and presents a significant more severe local inflammatory response than in patients with SBP [60, 61]. On the contrary, bacterascites is the term used to describe the colonization of ascitic fluid by bacteria in the absence of a local inflammatory reaction, which suggests the concurrent failure of defensive mechanisms [59]. 

The standard view of inflammation as a reaction to injury or infection might need to be expanded to account for the inflammatory processes induced by other types of adverse conditions [62]. Therefore, the splanchnic and systemic impairments that are produced during the evolution of the syndrome associated with PH could be considered of an inflammatory nature. If so, and similar to other types of inflammatory response, it would begin in the interstitial space [18] (Table 3). The early inflammatory response related to oxidative and nitrosative splanchnic damage, lipid peroxidation, and hypometabolism, could be associated with abnormal ion transport [1, 2]. There is increasing evidence that those conditions characterized by an intense inflammatory response have alterations in cellular membrane potential, with depolarization and abnormal ion transport [63]. Inflammatory mediators, which influence ion transport, are interleukins, tumor necrosis factor alpha (TNF-*α*), gamma-interferon (*γ*-IFN), bradykinin, and transforming growth factor (TGFs) [63, 64]. They trigger the release of specific messengers, like prostaglandins, nitric oxide, and histamine, which alter the function of the ion transport system through specific receptors, intracellular second messengers, and protein kinases [63]. 

The standard view of inflammation as a reaction to injury or infection might need to be expanded to account for the inflammatory processes induced by other types of adverse conditions [62]. Therefore, the splanchnic and systemic impairments that are produced during the evolution of the syndrome associated with PH could be considered of an inflammatory nature. If so, and similar to other types of inflammatory response, it would begin in the interstitial space [18] (Table 3). The early inflammatory response related to oxidative and nitrosative splanchnic damage, lipid peroxidation and hypometabolism, could be associated with abnormal ion transport [1, 2]. There is increasing evidence that those conditions characterized by an intense inflammatory response have alterations in cellular membrane potential, with depolarization and abnormal ion transport [63]. Inflammatory mediators, which influence ion transport, are interleukins, tumor necrosis factor alpha (TNF-*α*), gamma-interferon (*γ*-IFN), bradykinin and transforming growth factor (TGFs) [63, 64]. They trigger the release of specific messengers, like prostaglandins, nitric oxide and histamine, which alter the function of the ion transport system through specific receptors, intracellular second messengers and protein kinases [63]. 

In addition, disturbances of ion transport are produced in intra- and extracellular edema. It has been stated that small fluctuations in cell hydration or cell volume act as a potent signal for cellular metabolism and gene expression. Specifically, cell swelling triggers an anabolic signal [65]. Oxidative and nitrosative tissue damage could also increase lipid peroxidation with increased membrane permeability, increased degradation of extracellular matrix, and edema [66]. The accumulation of glycosaminoglycan fragments has been proposed as an important mechanism for edema formation due to its hydrophilic properties [67]. 

Glycosaminoglycans are long unbranched polysaccharide chains that tend to adopt highly extended random coil conformations and occupy a huge volume for their mass. They attract and entrap water and ions, thereby forming hydrated gels, while permitting the flow of cellular nutrients [67]. Under inflammatory conditions, hyaluronan, a nonsulphated glycosaminoglycan, is more polydisperse with preponderance to lowermolecular forms. Hyaluronan favors edematous infiltration of the tissues as well as the interstitial fluid flow and the tissue lymph pressure gradient [68]. Likewise, while the progression of interstitial edema reduces the blood capillary function, it simultaneously enhances lymphatic circulation (circulatory switch). 

Furthermore, splanchnic interstitial flow could be relevant for lymphangiogenesis [69]. The interstitial fluid flow associated with edema, even though it can be extremely slow, can have important effects on tissue morphogenesis and function, cell migration and differentiation and matrix remodeling, among other processes [70]. Abnormally increased interstitial flow rates can occur during low-grade inflammation and can also trigger fibroblasts to differentiate or remodel the extracellular matrix, thus contributing to the development of tissue fibrosis [70–74]. Interstitial flow may significantly alter the distribution of metalloproteinases and lymphatic growth factors inducing lymphatic endothelial cell migration and capillary morphogenesis [69, 72]. Moreover, upon splanchnic activation, mast cells, major effecter cells in host defense responses and immunity, release not only vasoactive substances, histamine, and serotonin, but also proteolytic enzymes favoring interstitial edema and remodeling [2]. Therefore, the substances which are released by the stress systems in the blood during PH, that is, aldosterone, renin, and catecholamines, will accumulate selectively in the splanchnic interstitial space because of an increased endothelial permeability [1]. Mast cells and lymphatic vessels are two important players in the development of the splanchnic inflammatory process. Thus, it has been shown that in vitro mast cells' degranulation impairs lymphatic contractile activity, probably through activation of H1 receptors by histamine. It has been suggested that this action could interfere with the expected ability of lymphatic vessels to reduce edema during inflammation [75]. 

Unidirectional fluid transport into the initial lymphatics from the splanchnic interstitial space could be facilitated by the primary and secondary valve systems and then by the contractile lymphatics [76, 77]. Increasing evidence suggests that lymphatic vessels might actively participate in the inflammatory process. Lymph flow is unidirectional from the lymphatic capillaries to larger collecting lymphatics. The forces that move the lymph along the collecting vessels include smooth muscle contraction. Also, the collecting lymphatic vessels are covered by smooth muscle cells and have intraluminal valves for preventing the backflow of lymph. This set of valves allows fluid to enter the lymphatic vessels, but not to escape. Oscillations in lymphatic pressure produced by periodic expansion and compression of the initial lymphatics cause the opening and closing of the primary valves, and consequently lymph formation [77]. During inflammation, the elevation of lymphatic endothelial permeability in an outward direction has two major effects. First, fluid is being cleared from the tissue less efficiently. With the increased permeability of the blood vessels already producing more fluid in the tissue than normal, the decreased transport by the lymphatics causes this edema to increase even more. Second, the leaking lymphatics allow inflammatory mediators to remain longer in the tissue therefore increasing the inflammatory intestinal response [77]. 

Mesenteric lymph transports cells (lymphocytes), lipids (chylomicrons), proteins (plasma proteins, immunoglobulins), enzymes (alkaline phosphatase, amylase), hormones (insulin) and electrolytes (chloride and bicarbonate) to the systemic circulation [76]. The biological role of the mesenteric lymph in the pathogenesis of splanchnic and systemic inflammation is not fully clarified up to date. However, there is now evidence showing that mesenteric lymph plays a key role in the pathogenesis of multiple organ dysfunction in trauma/hemorrhagic shock, burns, reperfusion injury, and surgical stress [16, 76, 78–80]. The hemodynamic alterations that PH imposes on the splanchnic circulation [6, 8] allow for considering that changes in the mesenteric lymph flow and composition are also produced. If so, the mesenteric lymph could play an etiopathogenic role in the multiple organ dysfunction developed in the portal hypertensive syndrome. Particularly, bacteria and toxins, like bacterial lipopolysaccharide (LPS) and inflammatory mediators with high affinity for chylomicrons, that is, lipids, could use the mesenteric lymph vessels to bypass the liver and induce a systemic response [81]. Worsening of the splanchnic portal hypertensive inflammatory response induced by hepatic dysfunction could increase the degree of oxidative/nitrosative and enzymatic stress in the interstitial space, favoring intestinal edema with mesenteric lymph flow and composition alterations. Lymphangiogenesis, the formation of new lymphatic vessels, occurs in several pathological conditions associated with chronic inflammation [82, 83], including portal hypertensive enteropathy [44] and liver cirrhosis [41, 42]. Inflammatory cells, through the secretion of stimulatory factors such as VEGF-C and TNF-*α*, can stimulate lymphatic endothelial cells [84–86]. Lymphangiogenesis generally accompanies angiogenesis [87], the basic structural alteration of the hypertensive portal gastroenteropathy [19, 20]. However, pathological lymphangiogenesis may occur in absence of blood vessels [86, 88]. The biological role of lymphangiogenesis in the pathogenesis of chronic inflammation needs further clarification. Inflammation triggers lymphangiogenesis [89–91] and may be beneficial for the resolution of chronic inflammation since lymphatic vessels remove inflammatory cells and mediators from the inflammation sites [90, 92]. Lymphovenous communications located both in lymphatic vessels and mesenteric lymph nodes [76] could also have a pathophysiological significance in the setting of chronic intestinal inflammation that needs to be explored.

One highly specialized form of tissue remodeling in chronic inflammation is lymphoid neogenesis, the development of new lymphoid tissue in inflammatory sites [93, 94]. It has been proposed that during chronic inflammation, lymphangiogenesis and lymphoid neogenesis would be synergistic processes that mutually amplify each other [94].

## 4. The Role of Mesothelial Cells in the Pathophysiology of the Portal Hypertensive Syndrome

**Figure 3:**
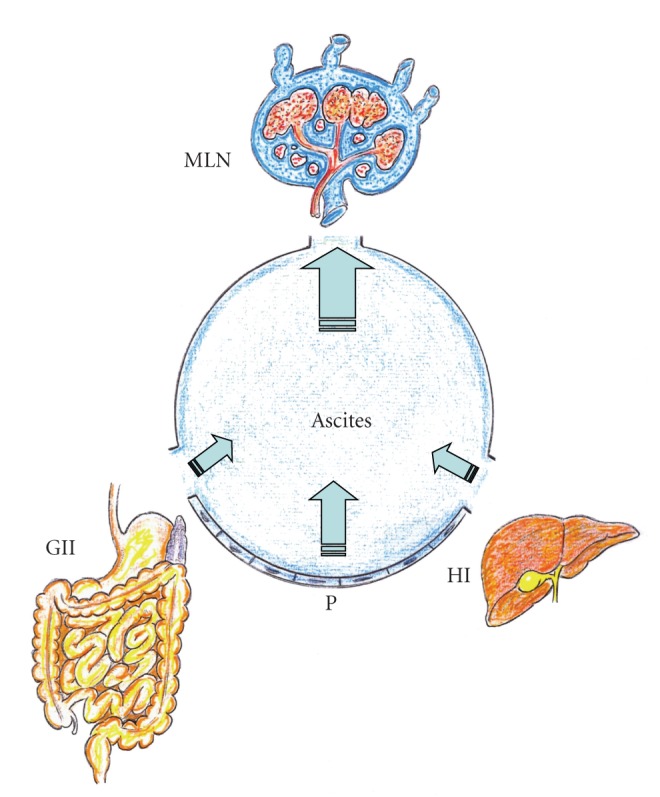
Acute-on-chronic splanchnic inflammation. Worsening of the portal hypertensive syndrome is associated with ascites. The ascitic fluid increases the lymphatic mesenteric flow as well as the lymphatic capillary stasis in the interstitium, which in turn would worsen the splanchnic inflammatory response. GII: Gastrointestinal interstitium; HI: Hepatic interstitium; MLN: Mesenteric lymph nodes; P: peritoneum.

Hepatic dysfunction related to fibrosis or cirrhosis would aggravate the grade of systemic inflammation characteristic of PH and as a result would increase the incidence of complications [1, 2]. Consequently, the vascular dysfunction or hyperdynamic systemic and splanchnic circulation, with increased mesenteric blood flow and portal pressure, would get worse, and interstitial hepatointestinal lymph flow would be favored. Splanchnic ischemia-reperfusion injury secondary to the acute vascular dysfunction could result in higher RAAS activity and excessive interstitial edema [47, 54, 95, 96]. The liver has been assumed to be the likely source of ascites in patients with liver disease [41]. Nevertheless, the cirrhotic liver is not the sole or even the major source of ascites in most patients [47, 54]. Weeping of fluid in great excess from the peritoneal lining and serosal surfaces of the bowel is often striking in PH as are dilated lymphatics on the surface of the small intestine, in the mesentery and within the retroperitoneal space [54]. Therefore, it is accepted that when lymphatic drainage mechanisms are overwhelmed, excess lymph is collected in the peritoneal cavity, thus causing ascites [50, 97, 98] (Figure 3). In summary, when PH is associated with severe liver disease, a higher decompensation of the splanchnic lymphatic system is induced since the lymphatic vessels' dilation plus the lymphangiogenesis do not have enough ability for draining the excessive lymph. The final consequence is the spilling of the remaining lymph into the peritoneal cavity with ascites which indeed in some cases does not have an efficient treatment.

Nevertheless, peritoneal mesothelial cells would not be considered as a passive barrier for the lymph leakage of splanchnic origin in ascites formation. In the normal peritoneal cavity, mesothelial cells play an important role as a source of intraperitoneal phospholipids. Demonstration of the hydrophobic nature and surface tension-reducing properties of this peritoneal secretion led to its comparison with pulmonary surfactant [99]. In addition, the overlapped peritoneal mesothelial cells have a surface covered with a great number of microvillus and a cytoplasm filled with a greater number of ribosomes, mitochondria, rough endoplasm reticulum, and Golgi apparatus [100]. Particularly, the numerous pinocytic vesicles in the membrane and the cytoplasm indicate active endo- exo- and transcytosis in the process of secretion and reabsorption of peritoneal fluid [100]. These characteristics of the peritoneal mesothelial cells suggest their active participation in the inflammatory response, as a developer in the intestinal wall in portal hypertensive syndrome, and therefore in ascites formation. 

Peritoneal mesothelial cells could participate in the splanchnic inflammatory response in experimental models of decompensated portal hypertensive syndromes, the same occurs in the extrahepatic cholestatic rat. Obstructive cholestasis is characterized by jaundice, discolored urine, pale stools, and pruritus [101]. The serious repercussions of cholestasis on the liver and on the systemic level have led to the creation of many experimental models so as to better understand its pathogenesis, prophylaxis, and treatment [102, 103]. Obstructive cholestasis causes cirrhotic chronic hepatic insufficiency and PH. Several surgical techniques for developing obstructive cholestasis have been described, especially in the rat. These techniques could be divided into two groups, macrosurgical and microsurgical. Macrosurgical techniques are based on the section of the common bile duct between ligatures. These macrosurgical techniques of obstructive extrahepatic cholestasis are called “bile duct ligation” (BDL) and cause the development of infected hilar biliary pseudocysts by dilation of the bile duct proximal end. As a result, an important number of the animals die during the first 2 weeks of the postoperative period because of sepsis caused by multiple abscesses in the intraperitoneal, hepatic, and pulmonary areas [103, 104]. 

**Figure 4:**
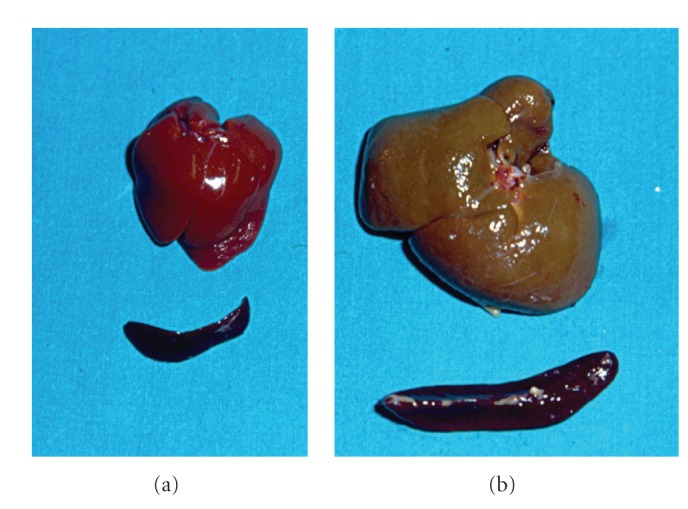
Liver and spleen in a sham-operated rat (a) and in a microsurgical extrahepatic cholestatic rat (b).

**Figure 5:**
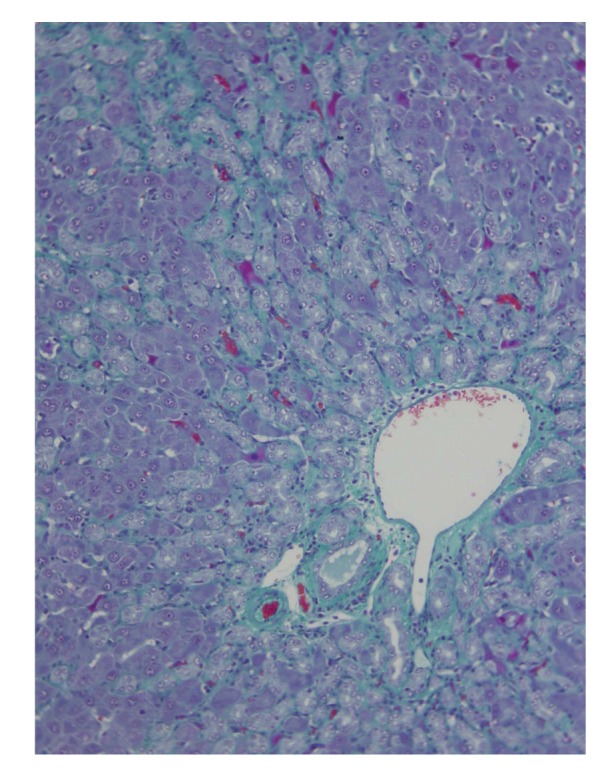
Liver fibrosis and biliary ductular proliferation after 6 weeks of microsurgical extrahepatic cholestasis in the rat (Masson, 100x).

To avoid these infectious complications, we have proposed performing a microsurgical technique which consists of the resection of the extrahepatic biliary tract [102, 103, 105]. The use of broad-spectrum antibiotics and vitamin K allows the long-term evolution of the rats [102, 103]. In the long-term evolution (8 to 10 weeks), microsurgical extrahepatic cholestatic rats develop hepatomegaly with a marked ductular proliferation and fibrosis [106] (Figure 4). It has been suggested that liver fibrogenesis resembles a wound-healing process leading to scar formation [107, 108]. The persistence of this inflammatory response through a longer evolution induces an “atypical” ductular proliferation with the development of a neuroendocrine compartment [109] (Figure 5). In relation to extrahepatic alterations, jaundice, choluria, PH with an enlarged spleen and collateral portosystemic circulation, hepatic encephalopathy, and ascites stand out [103, 110, 111]. Therefore, experimental extrahepatic cholestasis is a good model not only for studying chronic hepatic disease related to biliary obstruction, but also for studying extrahepatic complications, particularly ascites. 

In the rat, chronic liver disease secondary to obstructive cholestasis produces progressive hemodynamic dysfunction with ascites and hepatorenal syndrome. Bile duct-ligated rats after four weeks of biliary obstruction present an initial disturbance in renal function associated with ascites. Two weeks later, rats with obstructive cholestasis clearly developed hepatorenal syndrome with ascites [112]. In these chronic phases of macrosurgical obstructive cholestasis, high rates of bacterial translocation, with endotoxemia and consecutive systemic inflammatory response, also exist [113] (Table 4). In rats with obstructive cholestasis, the portal hypertensive syndrome with low-degree splanchnic and systemic inflammation can progress to severe systemic inflammatory response syndrome leading to multiple organ failure. After extrahepatic cholestasis, the rat can suffer the effects of generalized ischemia/reperfusion and exacerbation of oxidative and nitrosative stress [108]. It is accepted that there is a strong correlation between experimental obstructive jaundice and oxidative stress [114]. BDL mainly impairs the rat liver's ability of antioxidant regeneration, especially at the mitochondria level [115].

Thus, it has been demonstrated that treatment with antioxidants improves the hepatic cellular redox status [116, 117]. A decreased antioxidant capacity of the liver plays an important role not only in the pathogenesis of liver fibrosis or cirrhosis but also in the evolution of the portal hypertensive syndrome. In this hypothetical situation, during the evolution of the extrahepatic cholestatic rat, the progressive reduction of the hepatic antioxidant capacity is added to the initial PH with low-degree oxidative stress, which consequently represents a low-grade inflammatory state. Then, the intensity of the inflammatory response increases and adds severity to this syndrome [1, 2]. 

Long-term (6 weeks) microsurgical cholestatic rats show a splanchnic redistribution of cytokines, with an increase of Th1 (TNF-*α* and IL-1*β*) and Th2 (IL-4 and IL-10) cytokine production in the small bowel and in the mesenteric lymph nodes [118]. It has been proposed that this splanchnic inflammatory response associated with ascites could be mediated, among others factors, by mast cells [111]. Oxidative and nitrosative stress could be an important trigger of the splanchnic mast cell activation, which could be the cause of swelling, increased lymphatic flow, and production of peritoneal exudate [2]. In different conditions related to intestinal ischemia and oxidative stress, the mesenteric lymphatic circulation is more active than portal circulation for transporting inflammatory mediators released in the intestinal wall, endotoxins, and bacteria [119, 120]. This fact suggests that in other conditions that also produce these alterations, like severe portal hypertensive syndrome, the mesenteric lymph is a regional proinflammatory and antiinflammatory mediator vehicle, that is, a splanchnic one, but with a systemic origin [103]. 

The key pathophysiological role of the mesenteric lymph has been the reason for the development of adequate techniques to cannulate the rat mesenteric lymph duct [121–123]. 

The relative distribution profiles of protein functional classes in normal rodent mesenteric lymph differ significantly from that reported for plasma. The most abundant protein classes in mesenteric lymph are protease inhibitors, immune-related proteins, particularly those implicated in innate immunity, and carrier proteins [124]. Therefore, mesenteric lymph has a unique profile compared with plasma and thus represents more than a simple filtrate [124]. Recent proteomic analyses of posthemorrhagic shock mesenteric lymph have documented the increase of proteins functionally implicated in tissue inflammation [125]. These results provide a starting point for investigating in depth the pathophysiological role of mesenteric lymph in other conditions in which it is considered that the gastrointestinal tract is the engine of multiple organ dysfunction, like the portal hypertensive syndrome. Mesenteric lymph flow is generally believed to increase during intestinal inflammation. Although it is known that the mesenteric lymphatic system is intimately involved in and highly altered during the intestinal inflammation, the exact role of lymphatics is not yet known [126]. The mesenteric lymphatic system plays essential roles for transporting fluid, proteins, lipids, and immune cells. All these vital functions rely on the contractile and relaxation activities of the lymphatic vessel wall [127, 128]. Therefore, the release of inflammatory mediators in the intestinal interstitial space could play a pivotal role in modulating lymphatic vessel contractile activity. To this respect, mast cell intestinal hyperplasia in decompensated portal hypertensive rats could play a pivotal role in tissue swelling mediated by lymphatic contractile dysfunction. Increased interstitial pressure with lymphatic dysfunction in portal hypertensive gastroenteropathy could be also involved in the leakage of protein-rich lymph, causing a protein-losing enteropathy. The serum protein levels most affected by this process are those with limited ability to rapidly respond to such losses and generally have longer half-lives such as albumin [129]. If so, the hypoalbuminemia related with the cholestatic liver disease would be aggravated [103] which, in turn, would favor the production of interstitial edema. 

**Figure 6:**
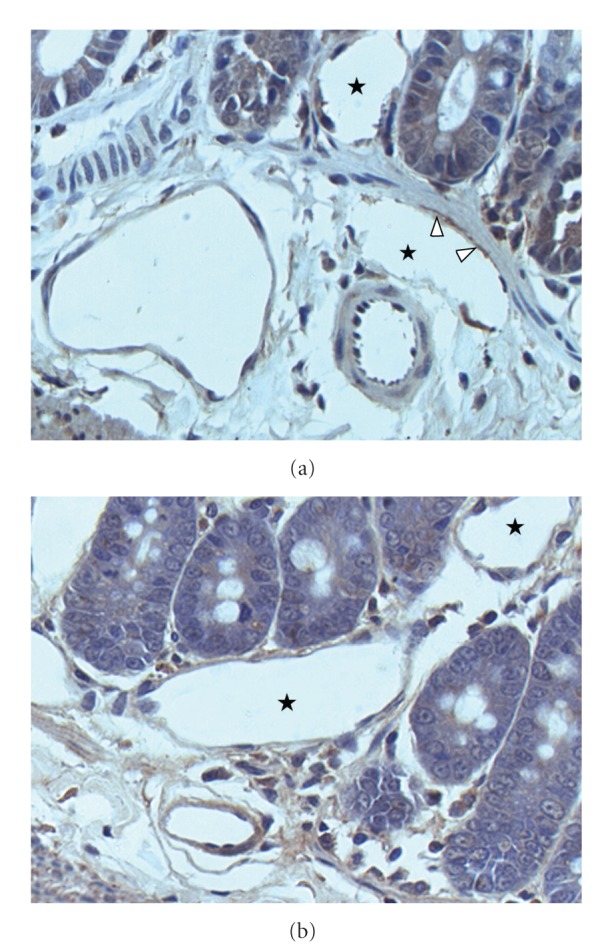
Intestinal lymphangiogenesis (a, b) is increased in rats with microsurgical extrahepatic cholestasis. ⋆ indicates lymphatic vessels lumen, and arrows indicate stained lymphatic endothelium. Immunohistochemical staining with Prox-1 antibody (×40).

**Figure 7:**
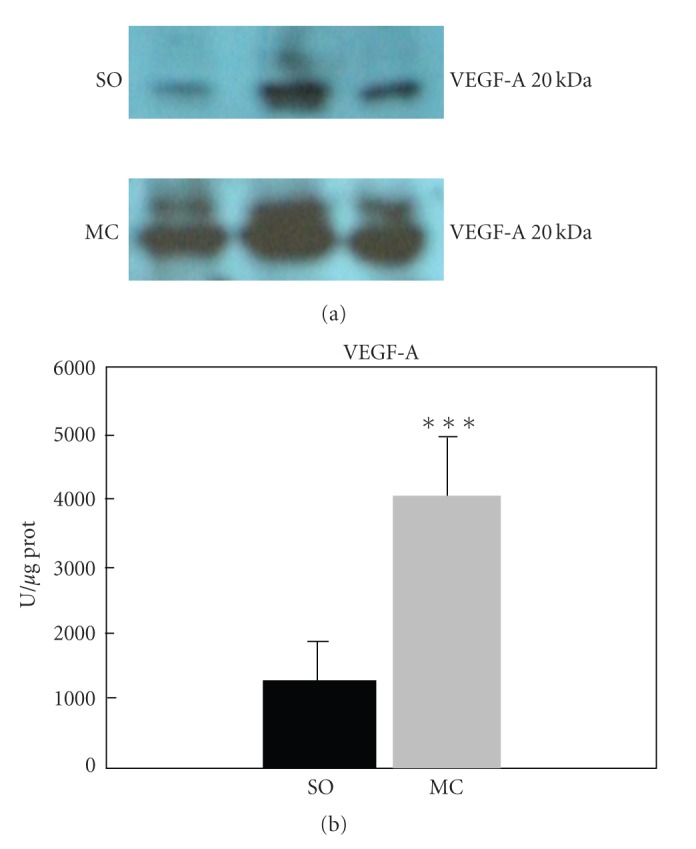
Increased intestinal levels of Vascular Endothelial Growth Factor (VEGF)-A in sham-operated (SO) and microsurgical microsurgical obstructive cholestatic rats (MC). The VEGF-A expression was assayed by Western blot and quantified by optical densitometry (O.D). ***P < .001: statistically significant value in relation with SO.

Using the transcription factor Prox 1, expressed in lymphatic endothelial cells, as a marker of lymphangiogenesis [130, 131], we have shown that in the intestinal mucosa/submucosa of rats with microsurgical cholestasis, a lymphatic hyperplasia is produced (unpublished results) (Figure 6). The associated expression of vascular endothelial growth factor (VEGF) in the small bowel in this experimental model suggests that this lymphatic growth factor could be an etiopathogenic factor of portal enteropathy, both due to the production angiogenesis and lymphangiogenesis [131] (Figure 7). 

Given that the splenic vein flows into the portal vein, any increase in portal pressure leads to an increase in the splenic venous pressure. This increase in the splenic venous pressure, in turn, increases intrasplenic fluid extravasation. As the splanchnic congestion worsens, intrasplenic microvascular pressure remains elevated and fluid efflux may overload the splenic lymphatic system, leading to an accumulation of excess fluid in the perivascular third spaces [132, 133]. Also in PH, a splenorenal and hepatorenal reflex-mediated reduction in renal vascular conductance has been described, which exacerbates sodium and water retention in the kidney and may eventually contribute to renal dysfunction [133]. Functional renal abnormalities that occur as a consequence of decreased effective arterial blood volume are responsible for fluid accumulation in the form of ascites and hepatic hydrothorax [45]. Ascites is the most common complication of PH and poses an increased risk for infections and renal failure [40, 45]. 

Contrary to prior belief, mesothelial cells that line the surfaces of the peritoneal cavity are not passive cells in ascites [134]. However, their ability to synthesize numerous cytokines, matrix proteins, intercellular adhesion molecules, and growth factors and their ability to present antigens to lymphocytes could play a critical role as immunomodulators during peritoneal injury and inflammation [134, 135]. The mesothelial response to PH associated with chronic liver diseases could be characterized by an acute-on-chronic inflammation that induces an increased vascular permeability, activation and expansion of the peritoneal macrophage and mast cell population, release of pro- and antiinflammatory mediators, increased matrix metalloproteinases, and tissue remodeling through subperitoneal angiogenesis and lymphangiogenesis. Peritoneal oxidative stress induced by chronic liver disease could induce acute mesothelial expression of proinflammatory, proangiogenic and profibrotic mediators as has been described in other peritoneal injury types [136]. Particularly, rat peritoneal mast cells (connective tissue mast cells) could play a main role in this hypothetical inflammatory process [137]. 

At last, ascites increases abdominal pressure, a continuum of pathophysiologic changes beginning with regional blood flow disturbance and culminating in frank end-organ failure and the development of Abdominal Compartment Syndrome [138]. Elevated intra-abdominal pressure compresses thin walled mesenteric veins promoting venous hypertension, intestinal interstitial edema, bacterial translocation, sepsis, and multiple organ failure [138, 139].

Bacterial translocation provides a mechanism for the pathogenesis of bacterial infections in experimental cholestasis [140]. Increased production of TNF-*α* may play an important role in the process of bacterial translocation in rats with cirrhosis and ascites because TNF-*α* blockade is able to downregulate it without increasing the incidence of systemic infections [141]. Jaundice is also an important mediator of the splanchnic and systemic inflammatory response in experimental models of cholestasis [108]. Bilirubin has a number of new and interesting biochemical and biological properties. In addition to having a protective role against oxidative stress, bilirubin has antiapoptotic and antimutagenic properties, as well as a strong role as an immune modulator [142]. Cholestatic jaundice also occurs in the setting of sepsis. Liver abnormalities in sepsis include cholestasis and hyperbilirubinemia. Hyperbilirubinemia particularly develops in sepsis in the setting of bacteriemia and precedes positive blood cultures in a third of all cases [143].

Besides ascites, serious complications such as SBP frequently ensue in decompensated cirrhosis [144]. SBP develops from the translocation of bacteria from the intestine, and successful management with early diagnosis and treatment with proper prevention in patients of high risk are necessary [144, 145]. Culture-negative neutrocytic ascites is considered to be a variant of SBP. The diagnosis of this variant is made when a patient has an elevated ascites fluid absolute polymorphonuclear leukocyte count, with a negative ascites fluid culture and no evident intra-abdominal surgically treatable source of infection [146]. Patients with ascitic fluid infection are prone to develop sepsis, severe sepsis, and septic shock [146, 147]. Interestingly enough, after surviving the first episode of ascitic fluid infection, hepatic failure and hypovolemic shock comprised a significant proportion of mortality, in addition to septic shock [146]. 

Ascites is the pathologic accumulation of fluid in the peritoneal cavity and is a common manifestation of liver failure, being one of the cardinal signs of PH [148]. Ascitic fluid formation is a not well-known pathogenic mechanism. However, ascitic fluid is a bioactive medium containing electrolytes, with high levels of sodium, proteins including albumin and enzymes, as well as cells including leukocytes [146]. Some of these characteristics make it similar to another bioactive medium, the amniotic fluid [149–151]. Amniotic fluid, the protecting liquid contained in the amnion cavity, is an essential component for fetal development and maturation during pregnancy [150, 152]. 

Body fluid is distributed among three major fluid spaces: intracellular fluid, interstitial fluid, and plasma. Nevertheless, the distribution of fluid in each of these compartments is dramatically different in the fetus compared to the adult [153]. Particularly, the amniotic fluid that surrounds the fetus may be considered an extension of the extracellular space of the fetus [152, 153]. Thus, the lymphatic system plays an essential role in the regulation of fluid distribution between the plasma and the interstitial fluid and probably with the amniotic fluid [153]. In patients with PH and ascites, it could also be hypothesized that the lymphatic splanchnic system plays a key role in the fluid distribution between the plasma and the ascitic fluid. If so, a pathological pathway of increasingly complex structures with a similar function to the management of the extracellular fluid would be formed.

**Figure 8:**
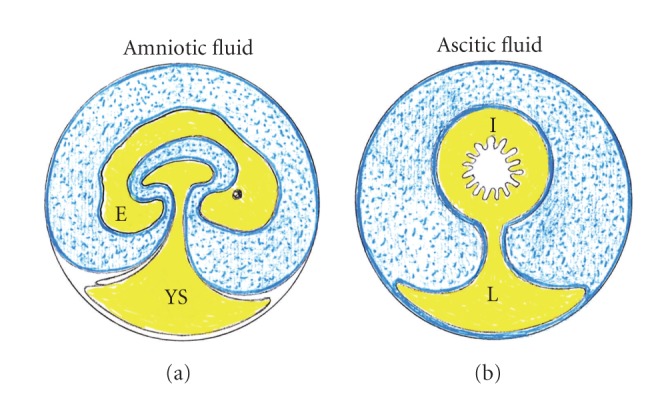
Comparative and schematic view of the amniotic egg and ascites in the decompensated portal hypertensive syndrome. Amniotic fluid circulates through the gastrointestinal tract of the embryo favoring its trophism and maturation. On the contrary, in the adult organism the proposed equivalent fluid, the ascitic fluid, is confined within peritoneal cavity. E: embryo; YS: yolk sac; I: intestine; L: liver.

At the early stages of pregnancy, amniotic fluid consists of a filtrate of maternal blood [151, 154]. That is why drugs taken by the mother can enter amniotic fluid by diffusion across the placenta during this period [151, 155]. However, its composition is known to change as pregnancy proceeds [149]. At these stages, amniotic fluid is a bioactive medium actively secreted by the cells lining the amniotic cavity, and as gestation progresses it includes significant volume of fetal urine [156]. The hypothetical comparison of the characteristics of amniotic and ascitic fluids would oblige raising again the role of peritoneal mesothelial cells in the etiopathogeny of ascites that occurs in the portal hypertensive syndrome (Figure 8). The first fluid to enter the gastrointestinal system is amniotic fluid, and it contributes to fetal nutritional requirements and plays a significant role in gut development and maturation [156]. Thus, growth-promoting effects of amniotic fluid are equivalent to human milk [156]. The trophic effect of orally consumed amniotic fluid is attributed in part to its content in growth factors, including epidermal growth factor (EGF), hepatocyte growth factor (HGF), transforming growth factor-alpha (TGF-*α*), fibroblast growth factor (FGF), insulin-like growth factor (IGF-s), and VEGF [156, 157].

The functional comparison of amniotic and ascitic fluids would imply that in the decompensated portal hypertensive syndrome the abdominal mesothelium acquires properties of the amniotic membranes or amnion. This hypothesis would imply several suppositions or suggestions. For example, the intestine in the case of portal hypertensive ascites could not benefit from the supposed trophic properties of the ascites fluid, given that the peritoneal cavity-gastrointestinal tract pathway doesn't exist. Likewise, the prenatal interruption of the amniotic fluid transit in cases of prenatal intestinal obstruction prevents the fetus from benefiting from its trophic properties, and it has been suggested that it contributes to fetal undergrowth [158]. 

The amniotic membrane is a tissue of particular interest because it possesses cells characteristic of stem cells with multipotent differentiation ability [159, 160]. Therefore, exploring the possibilities that the “ascitic peritoneum” would offer as a source of stem cells and growth factors could open new paths of knowledge for the study of its pathogeny. Even so, the ascitic fluid could be a source of powerful therapies for using its supposedly beneficial properties. The embryonic regression of the peritoneum that induces the inflammatory response induced by PH also could favor the presence of active antimicrobial components as has been described in the amniotic fluid [161]. Thus, the resistance to infection of the ascites would be explained, with usual subtle clinical manifestations in cases of secondary bacterial peritonitis [144, 145]. 

We have previously proposed the hypothesis that inflammation would represent the debut during the postnatal life of ancestral biochemical mechanisms that were used for normal embryonic development [1, 18]. The re-expression of these old mechanisms, with a prenatal solvent path, is perhaps inappropriate; they are anachronistic during postnatal life since they are established in a different environmental medium [162, 163]. That is why in the current paper, we propose the hypothetical existence of a regression to the prenatal functional mechanisms of the abdominal interstitium-lymphatic-mesothelium axis, in an attempt to integrate the existing knowledge of the portal hypertensive syndrome. In this hypothesis, it is considered that the main objective of this derepression of “dormant” biochemical mechanisms is the recuperation of the amnion. The major phylogenetic importance that this structure has perhaps lies in the fact that during development it protectively surrounds the embryo and creates a fluid-filled cavity in which the embryo develops [152]. Its acquisition, that is, the “amniotic egg” was one of the main causes that enabled amniotes to escape the bonds that confined their ancestors to aquatic environments [164, 165]. 

A poetic license could have been taken if we consider that the way to escape the sea is to take the sea with you, like an amniotic-mesothelial structure full of saline fluid. Since this ability would be associated in the postnatal life to mesothelial cells, its major spread through the body is not surprising; coating cavities (aracnoides, sinovial, pleura, testicular vaginal) to create virtual spaces that face trauma, infection or a tumor quickly carry out an exudative response. Therefore, although the mesothelial exudative inflammatory response is considered pathological, its proven evolutive and beneficial characteristics should not be forgotten from the embryonic, ontogenic, and phylogenetic points of view.

## 5. Conclusion

Ascites and hepatorenal syndrome are the major challenging complications of cirrhosis and portal hypertension that significantly affect the course of the disease. Particularly, the development of ascites severely impairs the quality of life and decreases the chance of survival. Obstructive cholestasis, which causes chronic hepatic insufficiency and PH with ascites, is a very useful experimental model for studying these complications. Several surgical techniques for developing obstructive cholestasis have been described, especially in the rat. The development of microsurgical techniques has made it easier to establish a new model of obstructive cholestasis in the rat that prevents the complications inherent to macrosurgical techniques based on BDL. 

The liver has been assumed to be the likely source of ascites in patients with liver disease, but the cirrhotic liver is not the sole or even the major source of ascites in most patients. Weeping of fluid in excess from the peritoneal lining and serosal surfaces of the bowel is often striking in PH as are dilated lymphatics on the surface of the small intestine, in the mesentery and within the retroperitoneal space. Therefore, it is accepted that when lymphatic drainage mechanisms are overwhelmed, excess lymph is collected in the peritoneal cavity, thus causing ascites. The mesothelial response to PH associated with chronic liver disease, could be characterized by an acute-on-chronic inflammation. In patients with PH and ascites, it could be hypothesized that the lymphatic splanchnic system in continuity with peritoneal mesothelial cells plays a key role in abnormal body fluid distribution resulting in ascites. The hypothetical comparison of the characteristics of amniotic and ascitic fluids would oblige raising again the role of peritoneal mesothelial cells in the etiopathogeny of ascites that occurs in the portal hypertensive syndrome. The functional comparison of amniotic and ascitic fluids could imply that in the decompensated portal hypertensive syndrome the abdominal mesothelium expresses properties similar to those of the amniotic membranes.

## Figures and Tables

**Table 1:** Splanchnic inflammatory phenotypes in the portal hypertensive syndrome.

(1) Ischemia-reperfusion phenotype
(i) Hyperdynamic blood circulation
(ii) Oxidative and nitrosative stress
(iii) Interstitial edema
(iv) Hyperdynamic lymphatic circulation
(2) Leukocytic phenotype
(i) Lymphatic immune dysfunction
(ii) Cell and bacterial interstitial translocation and activation
(iii) Interstitial enzymatic stress
(iv) Mesenteric adenitis
(v) Acute phase response
(3) Angiogenic phenotype
(i) Stromal remodeling
(a) Angiogenesis
(b) Lymphangiogenesis
(c) Fibrosis
(ii) Parenchymal remodeling
(a) Villous/crypt ratio decrease
(b) Goblet cell hyperplasia
(c) Hepatocytic steatosis

**Table 2:** Characteristics of progressive portal hypertensive syndrome.

(i) Increased:
(a) Hyperdynamic blood circulation
(b) Hypoxia
(c) Sodium and water retention
(d) Lymph formation
(e) Hyperdynamic lymphatic circulation
(ii) Lymph leakage into peritoneal cavity
(iii) Ascites
(iv) Refractory ascites
(v) Bacterascites
(vi) Spontaneous bacterial peritonitis
(vii) Secondary bacterial peritonitis
(viii) Hepatorenal syndrome
(ix) Sepsis
(x) Hypotension and hypothermia
(xi) Severe sepsis
(xii) Septic shock


**Table 3:** The *continuum* inflammatory splanchnic pathway of Portal Hypertension.

(i) Interstitium
(a) Oxidative and nitrosative damage
(b) Abnormal ion transport
(c) Degradation of extracellular matrix
(d) Edematous infiltration
(e) Immune cells activation
(ii) Mesenteric lymphatics
(a) Increased fluid flow
(b) Increased transport of immune cells
(c) Toxins and bacteria translocation
(d) Lymphangiogenesis
(iii) Peritoneal mesothelium
(a) Oxidative and nitrosative stress
(b) Abnormal ion transport
(c) Immune activation
(d) Enzymatic stress
(e) Lymph leakage
(f) Ascites

**Table 4:** Characteristics of the experimental cholestatic portal hypertensive syndrome.

(i) Severe hemodynamic dysfunction
(ii) Increased sodium and water retention
(iii) Interstitial dysfunction
(iv) Increased mesenteric lymph flow
(v) Bacterial translocation
(vi) Mesenteric adenitis
(vii) Endotoxemia
(viii) Negative acute phase response
(ix) Hypermetabolism
(x) Multiple organ dysfunction/failure
(a) Lung
(b) Central nervous system
(c) Kidney
(xi) Catabolism
(xii) Ascites
(xiii) Bacterial peritonitis
(xiv) Abdominal Compartment Syndrome
(xv) Sepsis
(xvi) Shock

## References

[1] Aller MA, Arias JL, Cruz A, Arias J (2007). Inflammation: a way to understanding the evolution of portal hypertension. *Theoretical Biology and Medical Modelling*.

[2] Aller MA, Arias JL, Arias J (2007). The mast cell integrates the splanchnic and systemic inflammatory response in portal hypertension. *Journal of Translational Medicine*.

[3] Cazzaniga M, Dionigi E, Gobbo G, Fioretti A, Monti V, Salerno F (2009). The systemic inflammatory response syndrome in cirrhotic patients: relationship with their in-hospital outcome. *Journal of Hepatology*.

[4] Malik R, Mookerjee RP, Jalan R (2009). Infection and inflammation in liver failure: two sides of the same coin. *Journal of Hepatology*.

[5] Aller MA, Nava M-P, Cuellar C (2007). Evolutive phases of experimental prehepatic portal hypertension. *Journal of Gastroenterology and Hepatology*.

[6] Iwakiri Y, Groszmann RJ (2006). The hyperdynamic circulation of chronic liver diseases: from the patient to the molecule. *Hepatology*.

[7] Rodríguez-Vilarrupla A, Fernández M, Bosch J, García-Pagán JC (2007). Current concepts on the pathophysiology of portal hypertension. *Annals of Hepatology*.

[8] Iwakiri Y, Groszmann RJ (2007). Vascular endothelial dysfunction in cirrhosis. *Journal of Hepatology*.

[9] Harrison DG, Widder J, Grumbach I, Chen W, Weber M, Searles C (2006). Endothelial mechanotransduction, nitric oxide and vascular inflammation. *Journal of Internal Medicine*.

[10] Llamas M-A, Aller MA, Marquina D, Nava M-P, Arias J (2010). Bacterial translocation to mesenteric lymph nodes increases in chronic portal hypertensive rats. *Digestive Diseases and Sciences*.

[11] Garcia-Tsao G, Albillos A, Barden GE, West AB (1993). Bacterial translocation in acute and chronic portal hypertension. *Hepatology*.

[12] Garcia-Tsao G, Wiest R (2004). Gut microflora in the pathogenesis of the complications of cirrhosis. *Best Practice and Research: Clinical Gastroenterology*.

[13] Moquillaza LM, Aller MA, Nava M-P, Santamaría L, Vergara P, Arias J (2010). Partial hepatectomy, partial portal vein stenosis and mesenteric lymphadenectomy increase splanchnic mast cell infiltration in the rat. *Acta Histochemica*.

[14] Cǎruntu FA, Benea L (2006). Spontaneous bacterial peritonitis: pathogenesis, diagnosis, treatment. *Journal of Gastrointestinal and Liver Diseases*.

[15] Prieto I, Aller MA, Santamaría L (2005). Prehepatic portal hypertension produces increased mast cell density in the small bowel and in mesenteric lymph nodes in the rat. *Journal of Gastroenterology and Hepatology*.

[16] Deitch EA (2002). Bacterial translocation or lymphatic drainage of toxic products from the gut: what is important in human beings?. *Surgery*.

[17] Puxeddu I, Ribatti D, Crivellato E, Levi-Schaffer F (2005). Mast cells and eosinophils: a novel link between inflammation and angiogenesis in allergic diseases. *Journal of Allergy and Clinical Immunology*.

[18] Aller MA, Arias JL, Nava MP, Arias J (2004). Post traumatic inflammation is a complex response based on the pathological expression of the nervous, immune and endocrine functional systems. *Experimental Biology and Medicine*.

[19] Viggiano TR, Gostout CJ (1992). Portal hypertensive intestinal vasculopathy: a review of the clinical, endoscopic, and histopathologic features. *American Journal of Gastroenterology*.

[20] Rondonotti E, Villa F, Signorelli C, de Franchis R (2006). Portal hypertensive enteropathy. *Gastrointestinal Endoscopy Clinics of North America*.

[21] Nagral AS, Joshi AS, Bhatia SJ, Abraham P, Mistry FP, Vora IM (1993). Congestive jejunopathy in portal hypertension. *Gut*.

[22] Misra V, Misra SP, Dwivedi M, Gupta SC (1997). Histomorphometric study of portal hypertensive enteropathy. *American Journal of Clinical Pathology*.

[23] Galli SJ, Kalesnikoff J, Grimbaldeston MA, Piliponsky AM, Williams CMM, Tsai M (2005). Mast cells as “tunable” effector and immunoregulatory cells: recent advances. *Annual Review of Immunology*.

[24] Diez-Arias JA, Aller MA, Palma MD (2001). Increased duodenal mucosa infiltration by mast cells in rats with portal hypertension. *Digestive Surgery*.

[25] Fernandez M, Mejias M, Angermayr B, Garcia-Pagan JC, Rodés J, Bosch J (2005). Inhibition of VEGF receptor-2 decreases the development of hyperdynamic splanchnic circulation and portal-systemic collateral vessels in portal hypertensive rats. *Journal of Hepatology*.

[26] Angermayr B, Mejias M, Gracia-Sancho J, Garcia-Pagan JC, Bosch J, Fernandez M (2006). Heme oxygenase attenuates oxidative stress and inflammation, and increases VEGF expression in portal hypertensive rats. *Journal of Hepatology*.

[27] Alonso MJ, Aller MA, Corcuera MT (2005). Progressive hepatocytic fatty infiltration in rats with prehepatic portal hypertension. *Hepato-Gastroenterology*.

[28] Aller MA, Vara E, García C (2006). Hepatic lipid metabolism changes in short- and long-term prehepatic portal hypertensive rats. *World Journal of Gastroenterology*.

[29] Sánchez-Patán F, Anchuelo R, Aller MA (2008). Chronic prehepatic portal hypertension in the rat: is it a type of Metabolic Inflammatory Syndrome?. *Lipids in Health and Disease*.

[30] Prieto I, Jiménez F, Aller MA (2005). Tumor necrosis factor-*α*, interleukin-1*β* and nitric oxide: induction of liver megamitochondria in prehepatic portal hypertensive rats. *World Journal of Surgery*.

[31] Tilg H, Diehl AM (2000). Cytokines in alcoholic and nonalcoholic steatohepatitis. *New England Journal of Medicine*.

[32] Alfter K, Von Kügelgen I, Haenisch B (2009). New aspects of liver abnormalities as part of the systemic mast cell activation syndrome. *Liver International*.

[33] Hotamisligil GS (2006). Inflammation and metabolic disorders. *Nature*.

[34] Nishimura S, Manabe I, Nagasaki M (2009). CD_8_
^  +^ effector T cells contribute to macrophage recruitment and adipose tissue inflammation in obesity. *Nature Medicine*.

[35] Popov Y, Schuppan D (2010). CD_8_
^  +^ T cells drive adipose tissue inflammation—a novel clue for NASH pathogenesis?. *Journal of Hepatology*.

[36] Rutkowski JM, Davis KE, Scherer PE (2009). Mechanisms of obesity and related pathologies: the macro- and microcirculation of adipose tissue. *FEBS Journal*.

[37] Harvey NL (2008). The link between lymphatic function and adipose biology. *Annals of the New York Academy of Sciences*.

[38] Pond CM (2005). Adipose tissue and the immune system. *Prostaglandins Leukotrienes and Essential Fatty Acids*.

[39] Bebenek M, Duś D, Koźlak J (2008). Fas expression in primary breast cancer is related to neoplastic infiltration of perilymphatic fat. *Advances in Medical Sciences*.

[40] Sanyal AJ, Bosch J, Blei A, Arroyo V (2008). Portal hypertension and its complications. *Gastroenterology*.

[41] Vollmar B, Wolf B, Siegmund S, Katsen AD, Menger MD (1997). Lymph vessel expansion and function in the development of hepatic fibrosis and cirrhosis. *American Journal of Pathology*.

[42] Greene RA, Dixon W (2002). Morphometric analysis of lymphatic vessels in primary biliary cirrhosis. *Hepatology Research*.

[43] Alexander JS, Ganta VC, Jordan PA, Witte MH (2010). Gastrointestinal lymphatics in health and disease. *Pathophysiology*.

[44] Ikeda R, Michitaka K, Yamauchi Y, Matsui H, Onji M (2001). Changes in gastrointestinal lymph and blood vessels in patients with cirrhotic portal hypertension. *Journal of Gastroenterology*.

[45] Cárdenas A, Arroyo V (2007). Management of ascites and hepatic hydrothorax. *Best Practice and Research: Clinical Gastroenterology*.

[46] Arroyo V, Fernandez J, Ginès P (2008). Pathogenesis and treatment of hepatorenal syndrome. *Seminars in Liver Disease*.

[47] Møller S, Henriksen JH, Bendtsen F (2008). Pathogenetic background for treatment of ascites and hepatorenal syndrome. *Hepatology International*.

[48] Mahmood G, Debnath CR, Mandal AK (2009). Evaluation of 100 cases of ascites. *Mymensingh Medical Journal*.

[49] Schrier RW, Arroyo V, Bernardi M, Epstein M, Henriksen JH, Rodes J (1988). Peripheral arterial vasodilation hypothesis: a proposal for the initiation of renal sodium and water retention in cirrhosis. *Hepatology*.

[50] Kashani A, Landaverde C, Medici V, Rossaro L (2008). Fluid retention in cirrhosis: pathophysiology and management. *QJM*.

[51] Salerno F, Cazzaniga M (2009). Autonomic dysfunction: often present but usually ignored in patients with liver disease. *Liver International*.

[52] Arroyo V, Gines P (1993). Mechanism of sodium retention and ascites formation in cirrhosis. *Journal of Hepatology*.

[53] Paulus BM, Ali S, Zia AA (2008). Causes and consequences of systemic venous hypertension. *American Journal of the Medical Sciences*.

[54] Witte CL, Witte MH (1983). Splanchnic circulatory and tissue fluid dynamics in portal hypertension. *Federation Proceedings*.

[55] Jiménez-Sáenz M, Soria IC, Bernardez JR, Gutierrez JM, Wong F, Blendis L (2003). Renal sodium retention in portal hypertension and hepatorenal reflex: from practice to science. *Hepatology*.

[56] Hamza SM, Kaufman S (2009). Role of spleen in integrated control of splanchnic vascular tone: physiology and pathophysiology. *Canadian Journal of Physiology and Pharmacology*.

[57] Cárdenas A, Ginès P (2009). Portal hypertension. *Current Opinion in Gastroenterology*.

[58] Riggio O, Angeloni S (2009). Ascitic fluid analysis for diagnosis and monitoring of spontaneous bacterial peritonitis. *World Journal of Gastroenterology*.

[59] Koulaouzidis A, Bhat S, Saeed AA (2009). Spontaneous bacterial peritonitis. *World Journal of Gastroenterology*.

[60] Zapater P, Francés R, González-Navajas JM (2008). Serum and ascitic fluid bacterial DNA: a new independent prognostic factor in noninfected patients with cirrhosis. *Hepatology*.

[61] Soriano G, Castellote J, Álvarez C (2010). Secondary bacterial peritonitis in cirrhosis: a retrospective study of clinical and analytical characteristics, diagnosis and management. *Journal of Hepatology*.

[62] Medzhitov R (2008). Origin and physiological roles of inflammation. *Nature*.

[63] Eisenhut M (2006). Changes in ion transport in inflammatory disease. *Journal of Inflammation*.

[64] Crowe SE, Luthra GK, Perdue MH (1997). Mast cell mediated ion transport in intestine from patients with and without inflammatory bowel disease. *Gut*.

[65] Häussinger D (1996). The role of cellular hydration in the regulation of cell function. *Biochemical Journal*.

[66] Kennett EC, Davies MJ (2007). Degradation of matrix glycosaminoglycans by peroxynitrite/peroxynitrous acid: evidence for a hydroxyl-radical-like mechanism. *Free Radical Biology and Medicine*.

[67] Jiang D, Liang J, Noble PW (2007). Hyaluronan in tissue injury and repair. *Annual Review of Cell and Developmental Biology*.

[68] Chen B, Fu B A model for charged molecule transport in the interstitial space.

[69] Ng CP, Helm C-LE, Swartz MA (2004). Interstitial flow differentially stimulates blood and lymphatic endothelial cell morphogenesis in vitro. *Microvascular Research*.

[70] Rutkowski JM, Swartz MA (2007). A driving force for change: interstitial flow as a morphoregulator. *Trends in Cell Biology*.

[71] Ng CP, Hinz B, Swartz MA (2005). Interstitial fluid flow induces myofibroblast differentiation and collagen alignment in vitro. *Journal of Cell Science*.

[72] Helm C-LE, Fleury ME, Zisch AH, Boschetti F, Swartz MA (2005). Synergy between interstitial flow and VEGF directs capillary morphogenesis in vitro through a gradient amplification mechanism. *Proceedings of the National Academy of Sciences of the United States of America*.

[73] Ng CP, Swartz MA (2006). Mechanisms of interstitial flow-induced remodeling of fibroblast-collagen cultures. *Annals of Biomedical Engineering*.

[74] Goldman J, Conley KA, Raehl A (2007). Regulation of lymphatic capillary regeneration by interstitial flow in skin. *American Journal of Physiology*.

[75] Plaku KJ, von der Weid P-Y (2006). Mast cell degranulation alters lymphatic contractile activity through action of histamine. *Microcirculation*.

[76] Fanous MYZ, Phillips AJ, Windsor JA (2007). Mesenteric lymph: the bridge to future management of critical illness. *Journal of the Pancreas*.

[77] Lynch PM, Delano FA, Schmid-Schönbein GW (2007). The primary valves in the initial lymphatics during inflammation. *Lymphatic Research and Biology*.

[78] Magnotti LJ, Xu D-Z, Lu Q, Deitch EA (1999). Gut-derived mesenteric lymph: a link between burn and lung injury. *Archives of Surgery*.

[79] Kaiser VL, Sifri ZC, Dikdan GS (2005). Trauma-hemorrhagic shock mesenteric lymph from rat contains a modified form of albumin that is implicated in endothelial cell toxicity. *Shock*.

[80] Watkins AC, Caputo FJ, Badami C (2008). Mesenteric lymph duct ligation attenuates lung injury and neutrophil activation after intraperitoneal injection of endotoxin in rats. *Journal of Trauma*.

[81] Wang Y, Ghoshal S, Ward M, de Villiers W, Woodward J, Eckhardt E (2009). Chylomicrons promote intestinal absorption and systemic dissemination of dietary antigen (ovalbumin) in mice. *PloS One*.

[82] Wilting J, Becker J, Buttler K, Weich HA (2009). Lymphatics and inflammation. *Current Medicinal Chemistry*.

[83] Bruyère F, Noël A (2010). Lymphangiogenesis: in vitro and in vivo models. *FASEB Journal*.

[84] Mouta C, Heroult M (2003). Inflammatory triggers of lymphangiogenesis. *Lymphatic research and biology*.

[85] Baluk P, Yao L-C, Feng J (2009). TNF-*α* drives remodeling of blood vessels and lymphatics in sustained airway inflammation in mice. *Journal of Clinical Investigation*.

[86] Jurisic G, Detmar M (2009). Lymphatic endothelium in health and disease. *Cell and Tissue Research*.

[87] Nakao S, Maruyama K, Zandi S (2010). Lymphangiogenesis and angiogenesis: concurrence and/or dependence? Studies in inbred mouse strains. *FASEB Journal*.

[88] Ji R-C (2009). Lymph node lymphangiogenesis: a new concept for modulating tumor metastasis and inflammatory process. *Histology and Histopathology*.

[89] Liersch R, Detmar M (2007). Lymphangiogenesis in development and disease. *Thrombosis and Haemostasis*.

[90] Cueni LN, Detmar M (2008). The lymphatic system in health and disease. *Lymphatic Research and Biology*.

[91] Vetrano S, Borroni EM, Sarukhan A (2010). The lymphatic system controls intestinal inflammation and inflammation-associated colon cancer through the chemokine decoy receptor D6. *Gut*.

[92] Shimamura K, Nakatani T, Ueda A, Sugama J, Okuwa M (2009). Relationship between lymphangiogenesis and exudates during the wound-healing process of mouse skin full-thickness wound. *Wound Repair and Regeneration*.

[93] Lai WK, Adams DH (2005). Angiogenesis and chronic inflammation; the potential for novel therapeutic approaches in chronic liver disease. *Journal of Hepatology*.

[94] Thaunat O, Kerjaschki D, Nicoletti A (2006). Is defective lymphatic drainage a trigger for lymphoid neogenesis?. *Trends in Immunology*.

[95] Kuiper JJ, Boomsma F, Van Buren H, De Man R, Danser AHJ, Van Den Meiracker AH (2008). Components of the renin-angiotensin-aldosterone system in plasma and ascites in hepatic cirrhosis. *European Journal of Clinical Investigation*.

[96] Bansal S, Lindenfeld JA, Sdirier RW (2009). Sodium retention in heart failure and cirrhosis. Potential role of natriuretics doses of mineralocorticoid antagonist?. *Circulation: Heart Failure*.

[97] Mayberry JC, Welker KJ, Goldman RK, Mullins RJ (2003). Mechanism of acute ascites formation after trauma resuscitation. *Archives of Surgery*.

[98] Dong MH (2008). Complications of cirrhosis. *Disease-a-Month*.

[99] Beavis J, Harwood JL, Coles GA, Williams JD (1994). Synthesis of phospholipids by human peritoneal mesothelial cells. *Peritoneal Dialysis International*.

[100] Obradovic MM, Stojimirovic BB, Trpinac DP, Milutinovic DD, Obradovic DI, Nesic VB (2001). Ultrastructure of peritoneal mesothelial cells. *Srpski Arhiv Za Celokupno Lekarstvo*.

[101] Li MK, Crawford JM (2004). The Pathology of Cholestasis. *Seminars in Liver Disease*.

[102] Aller MA, Mendez M, Nava MP, Lopez L, Arias JL, Arias J (2009). The value of microsurgery in liver research. *Liver International*.

[103] Aller MA, Prieto I, Cruz M, Aller MA, Arias J (2009). Extrahepatic cholestasis. *Microsurgery in Liver Research*.

[104] Aller MA, Duran M, Ortega L (2004). Comparative study of macro- and microsurgical extrahepatic cholestasis in the rat. *Microsurgery*.

[105] Aller MA, Lorente L, Alonso S, Arias J (1993). A model of cholestasis in the rat, using a microsurgical technique. *Scandinavian Journal of Gastroenterology*.

[106] Sánchez-Patán F, Anchuelo R, Corcuera M-T (2008). Biliary fibrosis in microsurgical extrahepatic cholestasis in the rat. *Microsurgery*.

[107] Ramadori G, Saile B (2004). Portal tract fibrogenesis in the liver. *Laboratory Investigation*.

[108] Aller MA, Arias JL, Garcia-Dominguez J, Arias JI, Duran M, Arias J (2008). Experimental obstructive cholestasis: the wound-like inflammatory liver response. *Fibrogenesis Tissue Repair*.

[109] Aller MA, Arias JL, Prieto I, Losada M, Arias J (2010). Bile duct ligation: step-by-step to cholangiocyte inflammatory tumorigenesis. *European Journal of Gastroenterology and Hepatology*.

[110] García-Moreno LM, Ángeles Aller M, Conejo NM (2002). Brain Ag-NOR activity in cholestatic rats with hepatic encephalopathy. *Hepatology Research*.

[111] Aller MA, Nava MP, Arias JL (2004). Microsurgical extrahepatic cholestasis in the rat: a long-term study. *Journal of Investigative Surgery*.

[112] Pereira RM, dos Santos RAS, Oliveira EA (2008). Development of hepatorenal syndrome in bile duct ligated rats. *World Journal of Gastroenterology*.

[113] Assimakopoulos SF, Vagianos CE (2009). Bile duct ligation in rats: a reliable model of hepatorenal syndrome?. *World Journal of Gastroenterology*.

[114] Wang G, Shen H, Rajaraman G (2007). Expression and antioxidant function of liver fatty acid binding protein in normal and bile-duct ligated rats. *European Journal of Pharmacology*.

[115] Portincasa P, Grattagliano I, Testini M (2007). Parallel intestinal and liver injury during early cholestasis in the rat: modulation by bile salts and antioxidants. *Free Radical Biology and Medicine*.

[116] Assimakopoulos SF, Vagianos CE, Patsoukis N, Georgiou C, Nikolopoulou V, Scopa CD (2004). Evidence for intestinal oxidative stress in obstructive jaundice-induced gut barrier dysfunction in rats. *Acta Physiologica Scandinavica*.

[117] Soylu AR, Umit H, Tezel A (2006). Antioxidants vitamin E and C attenuate hepatic fibrosis in biliary-obstructed rats. *World Journal of Gastroenterology*.

[118] García-Dominguez J, Aller MA, García C (2010). Splanchnic Th2 and Th1 cytokine redistribution in microsurgical cholestatic rats. *Journal of Surgical Research*.

[119] Carrico CJ, Meakins JL, Marshall JC (1986). Multiple-organ-failure syndrome. *Archives of Surgery*.

[120] Suliburk J, Helmer K, Moore F, Mercer D (2008). The gut in systemic inflammatory response syndrome and sepsis: enzyme systems fighting multiple organ failure. *European Surgical Research*.

[121] Waynforth HB, Flecknell PA, Waynforth HB, Flecknell PA (1992). Methods of obtaining body fluids. *Experimental and Surgical Techniques in the Rat*.

[122] Hauss DJ, Fogal SE, Ficorilli JV (1998). Chronic collection of mesenteric lymph from concious, tethered rats. *Contemporary Topics in Laboratory Animal Science*.

[123] Aller MA, Nava MP, Arias J, Aller MA, Arias J (2009). Techniques of blood, bile and lymph samples extraction. *Microsurgery in Liver Research*.

[124] Mittal A, Middleditch M, Ruggiero K (2008). The proteome of rodent mesenteric lymph. *American Journal of Physiology*.

[125] Fang J-F, Shih L-Y, Yuan K-C, Fang K-Y, Hwang T-L, Hsieh S-Y (2010). Proteomic analysis of post-hemorrhagic shock mesenteric lymph. *Shock*.

[126] Wu TF, MacNaughton WK, Von Der Weid P-Y (2005). Lymphatic vessel contractile activity and intestinal inflammation. *Memorias do Instituto Oswaldo Cruz*.

[127] Bohlen HG, Wang W, Gashev A, Gasheva O, Zawieja D (2009). Phasic contractions of rat mesenteric lymphatics increase basal and phasic nitric oxide generation in vivo. *American Journal of Physiology*.

[128] Wang W, Nepiyushchikh Z, Zawieja DC (2009). Inhibition of myosin light chain phosphorylation decreases rat mesenteric lymphatic contractile activity. *American Journal of Physiology*.

[129] Umar SB, Dibaise JK (2010). Protein-losing enteropathy: case illustrations and clinical review. *American Journal of Gastroenterology*.

[130] Hong Y-K, Detmar M (2003). Prox1, master regulator of the lymphatic vasculature phenotype. *Cell and Tissue Research*.

[131] Baluk P, McDonald DM (2008). Markers for microscopic imaging of lymphangiogenesis and angiogenesis. *Annals of the New York Academy of Sciences*.

[132] Jacobs-Kaufman S, Levasseur J (2003). Effect of portal hypertension on splenic blood flow, intrasplenic extravasation and systemic blood pressure. *American Journal of Physiology*.

[133] Hamza SM, Kaufman S (2009). Role of spleen in integrated control of splanchnic vascular tone: physiology and pathophysiology. *Canadian Journal of Physiology and Pharmacology*.

[134] Yung S, Chao TM (2009). Intrinsic cells: mesothelial cells—central players in regulating inflammation and resolution. *Peritoneal Dialysis International*.

[135] Di Paolo N, Nicolia GA, Garosi G (2008). The peritoneum: from histological studies to mesothelial transplant through animal experimentation. *Peritoneal Dialysis International*.

[136] Witowski J, Ksiazek K, Jörres A (2008). Glucose-induced mesothelial cell senescence and peritoneal neoangiogenesis and fibrosis. *Peritoneal Dialysis International*.

[137] Sick E, Niederhoffer N, Takeda K, Landry Y, Gies J-P (2009). Activation of CD47 receptors causes histamine secretion from mast cells. *Cellular and Molecular Life Sciences*.

[138] Cheatham ML (2009). Abdominal compartment syndrome: pathophysiology and definitions. *Scandinavian Journal of Trauma, Resuscitation and Emergency Medicine*.

[139] Ball CG, Kirkpatrick AW, McBeth P (2008). The secondary abdominal compartment syndrome: not just another post-traumatic complication. *Canadian Journal of Surgery*.

[140] Sztrymf B, Libert J-M, Mougeot C (2005). Cirrhotic rats with bacterial translocation have higher incidence and severity of hepatopulmonary syndrome. *Journal of Gastroenterology and Hepatology*.

[141] Francés R, Chiva M, Sánchez E (2007). Bacterial translocation is downregulated by anti-TNF-*α* monoclonal antibody administration in rats with cirrhosis and ascites. *Journal of Hepatology*.

[142] Moseley RH (1999). Sepsis and cholestasis. *Clinics in Liver Disease*.

[143] Marrero J, Martinez FJ, Hyzy R (2003). Advances in critical care hepatology. *American Journal of Respiratory and Critical Care Medicine*.

[144] Lee JM, Han K-H, Ahn SH (2009). Ascites and spontaneous bacterial peritonitis: an Asian perspective. *Journal of Gastroenterology and Hepatology*.

[145] Cárdenas A, Ginès P (2008). What’s new in the treatment of ascites and spontaneous bacterial peritonitis. *Current Gastroenterology Reports*.

[146] Kim SU, Kim DY, Lee CK (2010). Ascitic fluid infection in patients with hepatitis B virus-related liver cirrhosis: culture-negative neutrocytic ascites versus spontaneous bacterial peritonitis. *Journal of Gastroenterology and Hepatology*.

[147] Gustot T, Durand F, Lebrec D, Vincent J-L, Moreau R (2009). Severe sepsis in cirrhosis. *Hepatology*.

[148] Hou W, Sanyal AJ (2009). Ascites: diagnosis and management. *Medical Clinics of North America*.

[149] Cho C-KJ, Shan SJ, Winsor EJ, Diamandis EP (2007). Proteomics analysis of human amniotic fluid. *Molecular and Cellular Proteomics*.

[150] Tong X-L, Wang L, Gao T-B, Qin Y-G, Qi Y-Q, Xu Y-P (2009). Potential function of amniotic fluid in fetal development—novel insights by comparing the composition of human amniotic fluid with umbilical cord and maternal serum at mid and late gestation. *Journal of the Chinese Medical Association*.

[151] Gray T, Huestis M (2007). Bioanalytical procedures for monitoring in utero drug exposure. *Analytical and Bioanalytical Chemistry*.

[152] Schmidt W (1992). The amniotic fluid compartment: the fetal habitat. *Advances in Anatomy, Embryology, and Cell Biology*.

[153] Bellini C, Boccardo F, Bonioli E, Campisi C (2006). Lymphodynamics in the fetus and newborn. *Lymphology*.

[154] Brace RA (1997). Physiology of amniotic fluid volume regulation. *Clinical Obstetrics and Gynecology*.

[155] Koski KG, Fergusson MA (1992). Amniotic fluid composition responds to changes in maternal dietary carbohydrate and is related to metabolic status in term fetal rats. *Journal of Nutrition*.

[156] Wagner CL, Taylor SN, Johnson D (2008). Host factors in amniotic fluid and breast milk that contribute to gut maturation. *Clinical Reviews in Allergy and Immunology*.

[157] Moran ET (2007). Nutrition of the developing embryo and hatchling. *Poultry Science*.

[158] Lopez de Torre B, Tovar JA, Uriarte S, Aldazabal P (1992). The nutrition of the fetus with intestinal atresia: studies in the chick embryo model. *Journal of Pediatric Surgery*.

[159] Delo DM, De Coppi P, Bartsch G, Atala A (2006). Amniotic fluid and placental stem cells. *Methods in Enzymology*.

[160] Insausti CL, Blanquer M, Bleda P (2010). The amniotic membrane as a source of stem cells. *Histology and Histopathology*.

[161] Schlievert P, Johnson W, Galask RP (1976). Isolation of a low molecular weight antibacterial system from human amniotic fluid. *Infection and Immunity*.

[162] Aller MÁ, Arias JL, Nava MP, Arias J (2004). Evolutive trophic phases of the systemic acute inflammatory response, oxygen use mechanisms and metamorphosis. *Psicothema*.

[163] Aller MA, Arias JL, Sánchez-Patán F, Arias J (2006). The inflammatory response: an efficient way of life. *Medical Science Monitor*.

[164] Elinson RP, Beckham Y (2002). Development in frogs with large eggs and the origin of amniotes. *Zoology*.

[165] Blackburn DG, Flemming AF (2009). Morphology, development, and evolution of fetal membranes and placentation in squamate reptiles. *Journal of Experimental Zoology B*.

